# A Strategy to Model Nonmonotonic Dose-Response Curve and Estimate IC_50_

**DOI:** 10.1371/journal.pone.0069301

**Published:** 2013-08-01

**Authors:** Hui Zhang, Jeanne Holden-Wiltse, Jiong Wang, Hua Liang

**Affiliations:** 1 Department of Biostatistics, St. Jude Children's Research Hospital, Memphis, Tennessee, United States of America; 2 Department of Biostatistics and Computational Biology, University of Rochester Medical Center, Rochester, New York, United States of America; 3 Department of Medicine and Microbiology and Immunology, University of Rochester Medical Center, Rochester, New York, United States of America; University of Missouri, United States of America

## Abstract

The half-maximal inhibitory concentration IC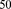 is an important pharmacodynamic index of drug effectiveness. To estimate this value, the dose response relationship needs to be established, which is generally achieved by fitting monotonic sigmoidal models. However, recent studies on Human Immunodeficiency Virus (HIV) mutants developing resistance to antiviral drugs show that the dose response curve may not be monotonic. Traditional models can fail for nonmonotonic data and ignore observations that may be of biologic significance. Therefore, we propose a nonparametric model to describe the dose response relationship and fit the curve using local polynomial regression. The nonparametric approach is shown to be promising especially for estimating the IC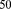 of some HIV inhibitory drugs, in which there is a dose-dependent stimulation of response for mutant strains. This model strategy may be applicable to general pharmacologic, toxicologic, or other biomedical data that exhibits a nonmonotonic dose response relationship for which traditional parametric models fail.

## Motivation

Drugs that inhibit the reverse transcriptase (RT) activity of Human Immunodeficiency Virus (HIV) are widely used to treat HIV infection. RT is an ideal target for antiviral HIV therapy because it is the key required for HIV replication. Non-nucleoside reverse transcriptase inhibitors (NNRTIs) inhibit RT activity by selectively binding RT at a hydrophobic binding pocket adjacent to the polymerase active site. Efavirenz (EFV) is an commonly used NNRTI to treat HIV infection [1]–[3] but patients can develop resistance to this drug because of the development of mutations in the NNRTI binding cite which in turn inhibits NNRTI binding [4] and can lead to resistance mutations such as K101E, K103N, Y188C, G190S, G190A [5], [6], and L100I [7].

Understanding the pharmacodynamic properties associated with the development of NNRTI resistant mutations is vital for devising treatment strategies for HIV. In pharmacodynamics, the drug-target interaction can be modeled by:
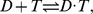
where 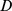 denotes the drug and 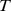 the target (usually enzyme). Drug efficiency is primarily determined by the drug target binding affinity. In pharmacodynamic studies, the drug target affinity is usually assessed by comparing dose response curves: the stronger the drug binds target, the steeper the curve is.

Therefore, a critical index of the dose response curve, the half-maximal inhibitory concentration (IC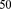), is commonly used to compare the binding affinities of drugs to the same target. IC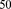 represents the concentration of a drug that is required for 50% of maximal inhibition in vitro. IC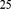 and IC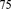 are the concentrations corresponding to 25% and 75% inhibition, respectively. The dose response curve usually has the steepest portion in the middle. Thus, using IC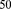, rather than IC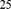 or IC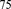, minimizes the random error for estimation, making IC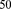 the preferred measure of drug affinity.

To estimate the IC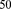 value, several nonlinear functions have been commonly used, for example,
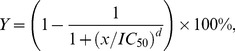
(1)where 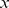 is the drug concentration, 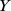 is the percentage of inhibition at this concentration, and 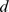 is a shape parameter. Other parametric models include the complementary log-log model for asymmetric quantal response data, and the two-parameter Weibull model for carcinogenic experiments [8].

**Figure 1:**
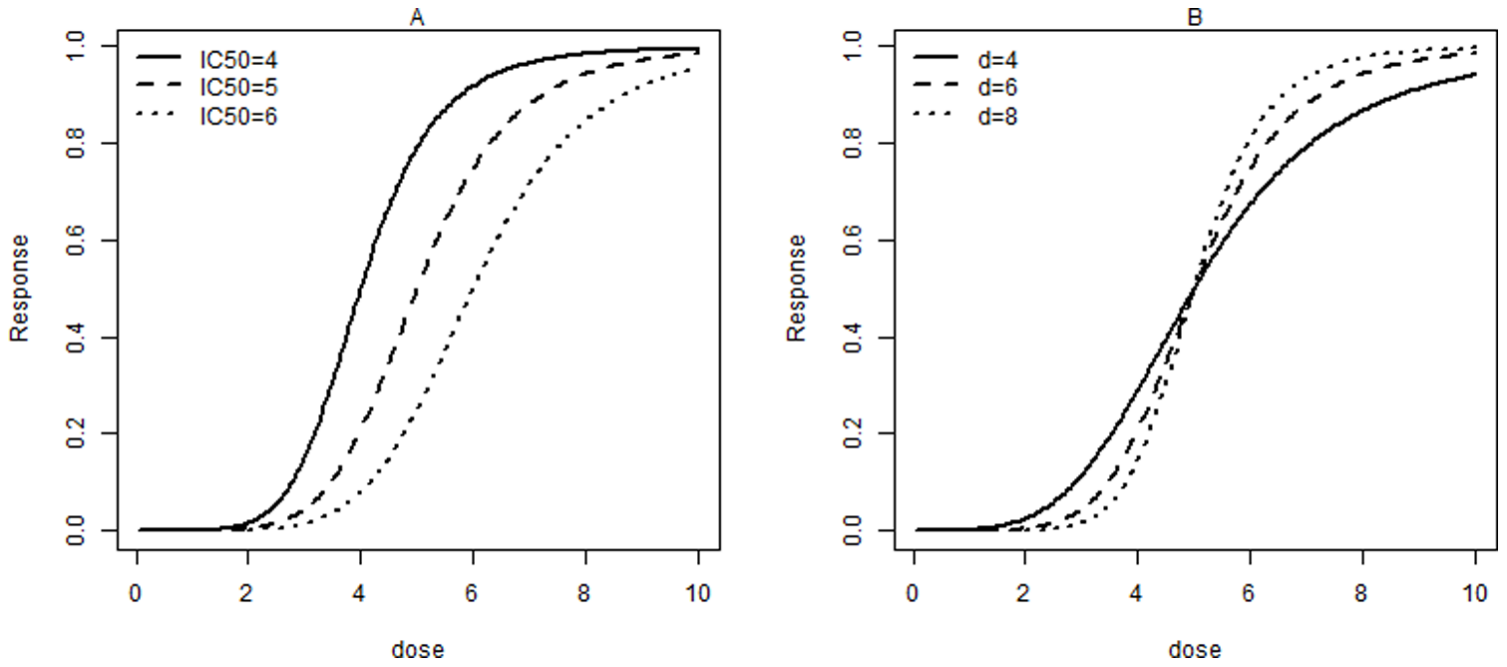
Dose response curves for various IC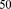 (A) or shape parameters, 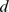 (B).

An important feature of the function (1) is that as the value of 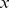 increases from 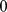 to infinity, 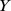 increases from 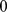 to 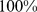, reflecting that a drug's inhibitory potential changes from none to full inhibition as the drug concentration increases (Fig. 1A). As per (1), a steeper dose response curve corresponds to a smaller IC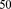; for a given IC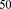, the curve shape is depicted by 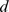 (Fig. 1B). In addition, this function curve has the steepest portion in the middle, which is a characteristic of a sigmoidal dose response relationship.

The advantages of this function are that (a) it is symmetrically about the IC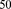; (b) it is monotonic, which equals that the target protein has an inhibitory binding site only, and the drug has an inhibitory effect only; and (c) IC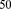 and 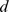 can be easily estimated under certain conditions. On the other hand, these advantages become restrictive when some conditions fail, for instance, if observations are not monotonic. As a consequence, the models can produce remarkably biased estimates or not even fit the observations. For example, Bliss's beetle data show that symmetry is not a required feature of a dose response curve [9]. Another example is that a viral mutation occurs before the drug concentration reaches a certain level, such as in the following example.

**Figure 2:**
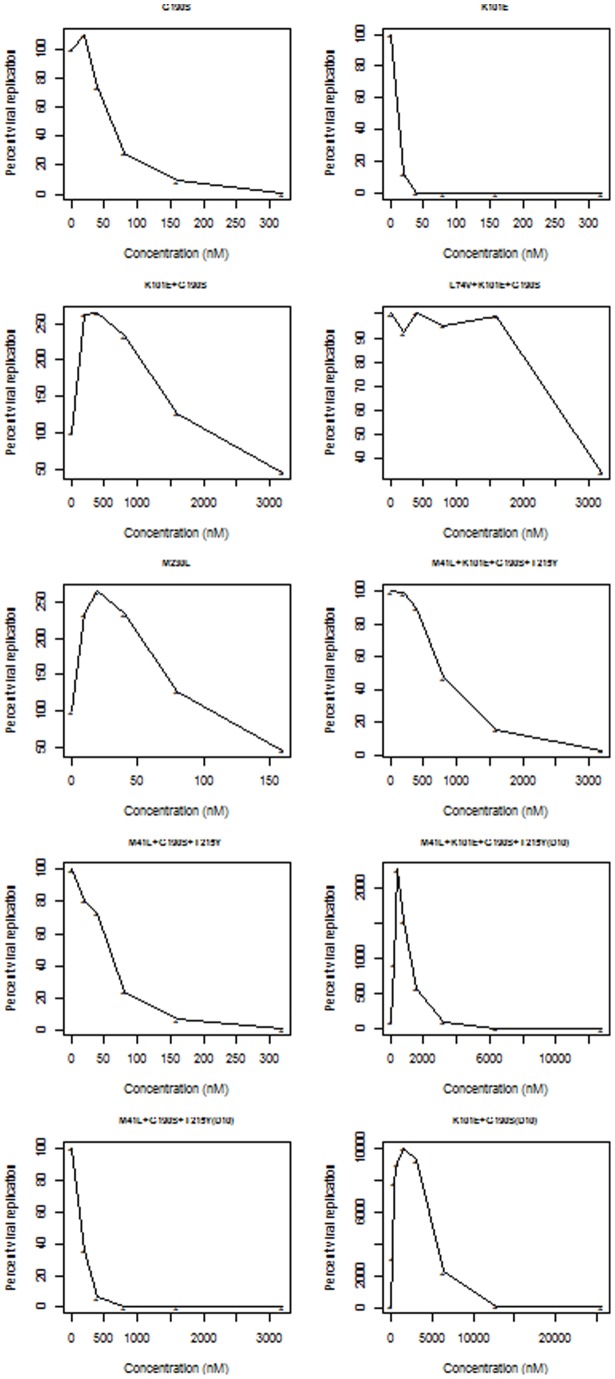
Dose response curves for viral replication of various HIV mutations at different EFV concentrations. The HIV strain names are the same as in previous publication [11]. Dose response was determined as proportion reduction in HIV replication at a given EFV dose relative to HIV replication in the absence of EFV. Viral replication above 100% indicates that suboptimal doses of EFV potentiate the ability of the viral strain to replicate compared to the absence of EFV. The data used to generate figures is available upon request.

A recent study on HIV mutations conferring resistance to NNRTI found that the monotonicity relation does not always hold [10]. The dose response was determined as proportion reduction in HIV replication at a given NNRTI dose relative to viral replication in the absence of drug. As seen in Fig. 2, the study shows that replication of HIV mutation M230L was promoted when the concentration of EFV is lower than 70 nM. Similarly, our data example shows that increasing the concentration of EFV stimulates the replication of HIV K101E+G190S mutant strain when EFV concentrations are below 2000 nM (Fig. 2). It has also been reported that EFV stimulation of the K101E+G190S double mutant strain can be abolished by the presence of additional M41L+T215Y mutation [11]. A potential explanation for this nonsigmoidal dose response relationship is that dimerization is essential for a fully functional RT. For the double mutant K101E+G190S strain, at low concentrations EFV can enhance the dimerization of the two subunits of RT without interfering with the binding of the incoming nucleotide during DNA polymerization.

As the real dose response relationship is nonmonotonic in our example, our preliminary analysis indicates that traditional estimation methods for the sigmoidal model (1) fitting lead to results that either do not converge or are remarkably biased, which may reach an erroneous conclusion. Thus, appropriate estimation of IC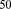 for this type of dose response relationship poses statistical challenges. If we use model (1) to fit data when the dose response pattern is nonmonotonic, the fit is poor, and the estimated IC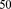 values are not reliable because the fact that lower EFV concentrations can enhance replication of an HIV mutant strain is ignored.

Thus, to appropriately estimate the pattern of observations and then estimate IC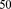, we developed a robust modeling strategy to test whether: (i) our model fitting is comparable to monotonic parametric models such as model (1) when the observed data are monotonic; and (ii) our model fitting yields reasonable estimates when the data pattern is nonmonotonic and monotonic parametric models, such as model (1), does not work.

The rest of this paper is organized as follows. Section 2 briefly introduces monotonicity testing, our model, estimation, and test methods. Section 3 gives simulation results, including p-values of the monotonicity test. Section 4 presents extensive analysis of our real data example, including estimated IC
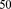
s using the proposed model and model (1) when it is appropriate.

## Methods

We propose that the inhibition percentage 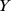 and concentration 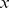 are related in the form
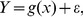
(2)where 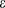 is the measurement error with mean zero and finite variance, 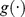 is a mathematical function, but no restrictions are applied on the form of 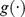 (i.e., no specified 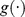 as quadratic, parametric, or increasing in 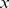, etc.) Since the structure of the model is not fixed, it is called a nonparametric method. Hence, we perform an empirical analysis of the data to estimate 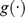. We use the observations 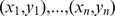 to estimate 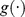, denoted as 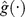, by appropriate statistical techniques. The IC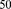 may be estimated as the point 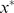 that satisfies 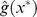 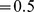. As noted above, we first need to determine whether the function 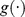 is monotonic.

### Subsection 1 Monotonicity test

Testing the monotonicity of a dose response relationship is of practical interest and has been studies previously. Several parametric and nonparametric methods have been proposed in the statistical literature. For example, Ramsay [12] studied the use of monotone splines to model a dose response function. Bowman et al. [13] developed a monotonicity test by using local linear estimation of the curve, followed by a critical bandwidth test. Hall and Heckman [14] proposed an alternative approach that focuses on “ running gradient” estimation over very short intervals. The method of Hall and Heckman is more effective in estimating the flat part of the curve and is also more sensitive to small dips in the curve.

For our study, we adopt the method of Hall and Heckman, which is based on the following principle. Let 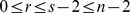 be integers and 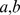 be constants. For each pair of 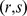, define the estimators of 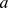 and 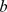 by 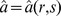 and 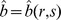, as the arguments of the following objective function:
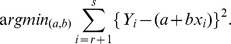



Define
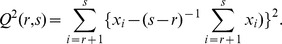



Let 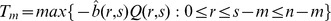, where 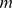 satisfies 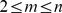. Hall and Heckman [14] stated that a large 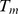 indicates that the null hypothesis, 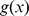 being monotonic, should be rejected. To obtain the 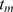 on the basis of 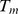, they suggested the following procedure. First, data should be fit with the nonparametric model 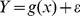. An estimation of 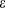 should be obtained by a consistent estimator of 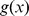, such as the local linear estimator. Assuming that a constant function is the most difficult nondecreasing form to be tested, Hall and Heckman used 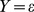 to obtain the 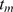. Specifically, using the estimated 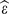, they resampled and obtained a new dataset 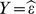, by which they obtained 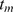. Repeated sampling n times resulted in a set 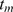 of size 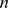. By taking the 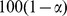
th ordered 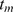
s as the critical value for 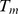, that is, when 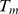 obtained from the real data is greater than this 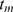, we claim that the function is nonmonotonic at the 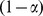 level.

### Subsection 2 Nonparametric fitting

We used local linear regression [15] to fit the dose response curve. Assuming that 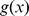 has bounded, continuous second partial derivatives, by Taylor expansion, 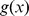, in a neighborhood of 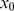, can be approximated as: 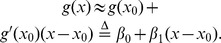 The estimator of 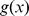 at 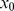 is the solution of 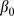 by minimizing 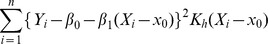 subject to (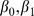), where 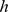 is a bandwidth controlling the size of the local neighborhood, 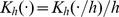, with 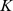 being a kernel function assigning weights to each data point.

## Simulation

To investigate how the Hall and Heckman [14] test performed for small and moderate sample sizes, we conducted a simulation study. Let 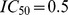 and 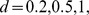 or 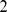, 
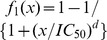
 for 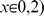. Consider 2 cases: (a) 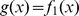; and (b) 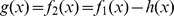, where 
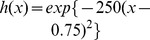
.

**Figure 3:**
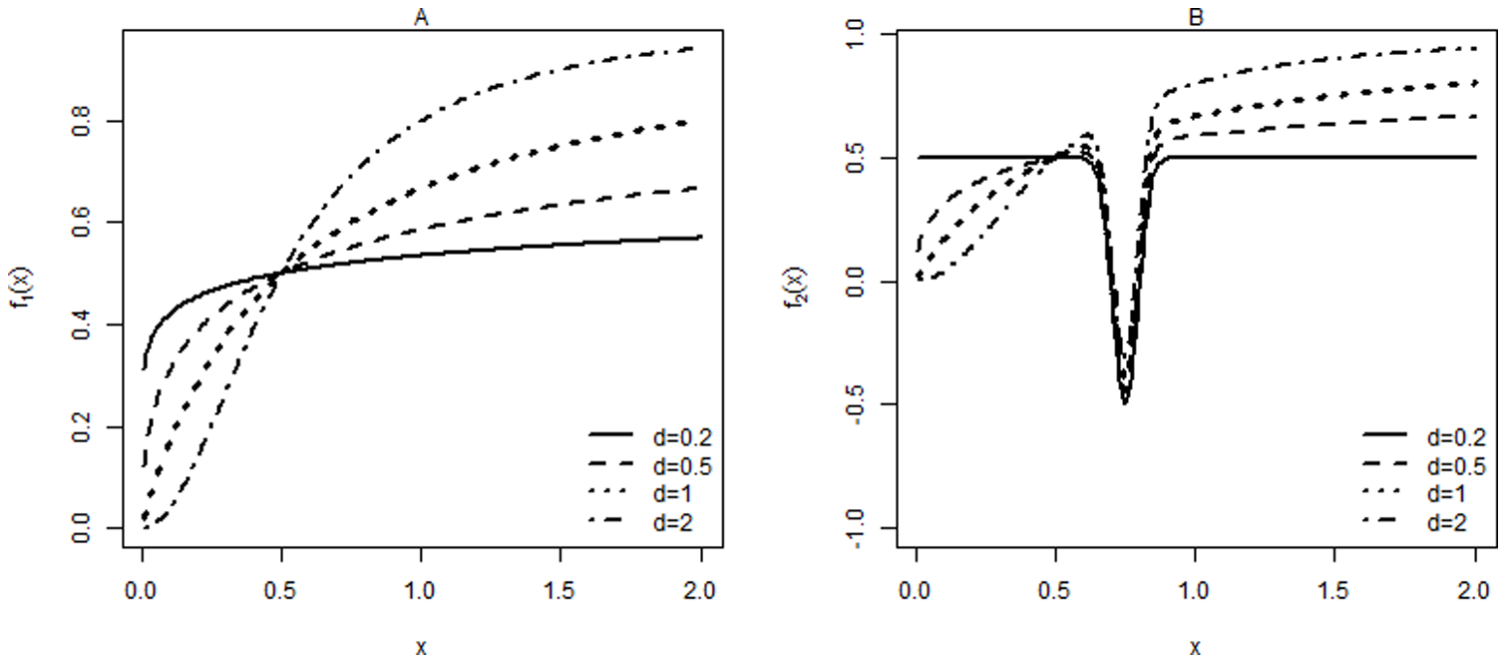
The patterns of functions 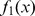, (A); and 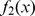, (B); for different 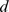 values.

Case (a) reflects that 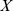 and 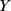 have a monotonic relationship, whereas case (b) indicates that the monotonicity is violated. Case (b) is based on the relationship between the inhibition of HIV mutant K101E+G190S strain and EFV concentration from our real data example, and is meant to show the performance power of the test. Figure 3 depicts the patterns of 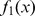 and 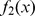, with 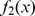 showing a pronounced dip around 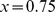.

The error 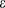 follows a normal distribution 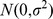, with 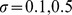. Our data are generated from the model (2) with 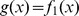 or 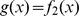, and 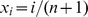. For each case, we considered 3 sample sizes 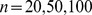, and generated 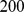 independent datasets for each of the 3 error variances.


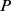
-value was determined as the probability that 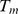 is greater than 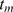. Table 1 shows the 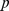-values of different simulation cases. As 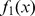 is a nondecreasing function, the 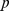-values should be greater than 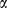, which was set at 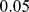; in contrast, 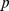-values for 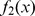 should be lower than 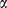. All test results based on 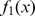 gave 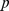-values greater than 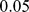, although there was a slight trend of decreasing 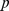-values as 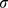 increases. Thus, on the basis of 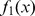 simulations, the monotonicity test method did not reject the null hypothesis at 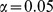 and correctly concluded that the relationship is monotonic. In contrast, when 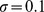, all test results based on 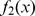 showed 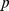-values lower than 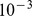, indicating that the null hypothesis (monotonicity) would be correctly rejected even when the sample size is very small. When 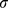 was increased to 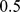 and the sample size was as small as 20, smaller 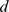 values, i.e. higher dip sizes (Fig. 3), still gave 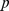-values lower than 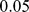. However, greater 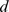 values showed 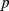-values slightly higher than 0.05. When the sample size was increased to 50, all 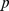-values were lower than 0.05. When the sample size was further increased to 100, all 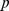-values were lower than 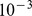. These results indicate that even with a high noise level, the monotonicity test is still reliable, particularly for large sample sizes. These results show that the monotonicity test is reliable and robust.

**Table 1:** The 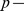
value (standard deviation) of the monotonicity tests for the simulation study.

		g(x) = f_1_(x)			g(x) = f_2_(x)		
σ	d	n = 20	n = 50	n = 100	n = 20	n = 50	n = 100
0.1	0.2	0.195(0.349)	0.201(341)	0.303(0.401)	*	*	*
	0.5	0.373(0.447)	0.364(0.425)	0.440(0.452)	*	*	*
	1	0.541(0.474)	0.595(0.447)	0.633(0.428)	*	*	*
	2	0.723(0.414)	0.720(0.413)	0.787(0.382)	*	*	*
0.5	0.2	0.119(0.287)	0.111(0.273)	0.095(0.249)	0.030(0.143)	0.001(0.016)	*
	0.5	0.091(0.255)	0.149(0.309)	0.179(0.337)	0.022(0.113)	0.006(0.072)	*
	1	0.225(0.384)	0.278(0.403)	0.229(0.367)	0.079(0.240)	0.026(0.141)	*
	2	0.298(0.411)	0.322(0.419)	0.397(0.429)	0.113(0.278)	0.034(0.164)	*

* indicates that both 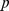-value and its associated standard deviation are less than 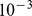.

## Real Data Analysis

We performed a monotonicity test for all HIV mutation dose response curves for percent viral replication compared to no drug (Fig. 2). For each mutant strain, we repeated the monotonicity test 100 times and have reported the average 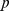-value in Table 2.

**Table 2:** The average 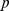-values of the monotonicity test for the real data example.

HIV strain	G190S	K101E	K101E+G190S	L74V+K101E+G190S	M41L+K101E+G190S+T215Y
*p*-value	1	0.9438	0.007	0.712	1

HIV strain	M230L	M41L+G190S+T215Y	K101E+G190S(D10)	M41L+G190S+T215Y(D10)	M41L+K101E+G190S+T215Y(D10)
*p*-value	<10^−3^	1	<10^−3^	0.984	0.397

The null hypothesis of monotonicity in HIV mutants K101E+G190S, M230L, and K101E+G190S(D10) was rejected with 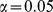 (Table 2). This result is consistent with the observed shape of the dose response curves (Fig. 2). Surprisingly, the test failed to reject 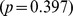 the monotonicity hypothesis for M41L+K101E+G190S+T215Y(D10) data, probably because the local polynomial fitting of this dataset still gives a non decreasing curve.

We then used the traditional sigmoidal model to fit the data and found that this method did not converge for HIV mutants K101E+G190S, M230L, and K101E+G190S(D10) because of the nonmonotonicity while the model did converge for datasets G190S, K101E, L74V+K101E+G190S, M41L+K101E+G190S+T215Y, M41L+G190S+.

**Figure 4:**
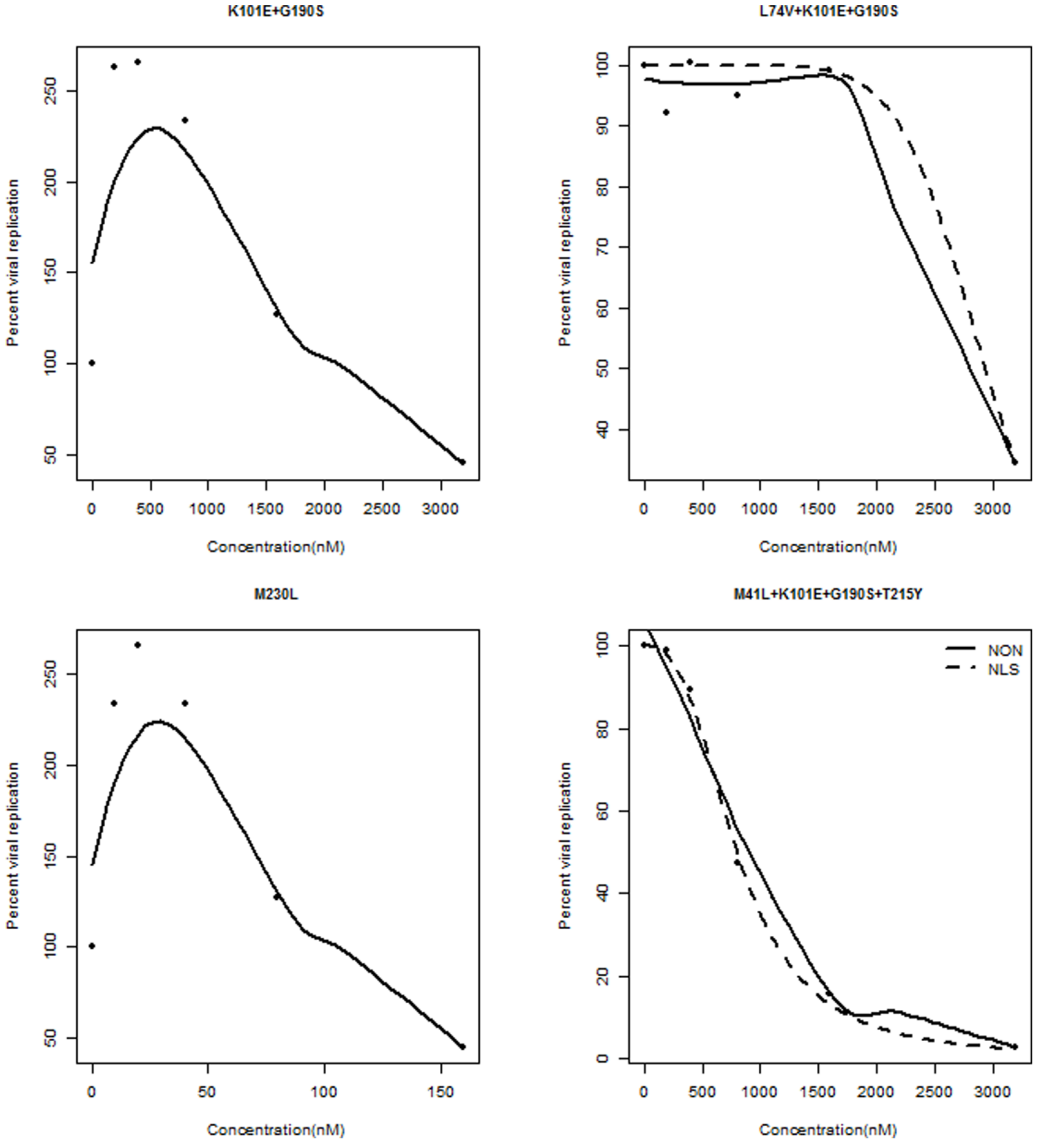
The fitted curves. Solid line indicates the nonparametric model and dashed line indicates the sigmoidal model if it is available.

T215Y, M41L+K101E+G190S+T215Y(D10), and M41L+G190S+T215Y(D10). We then fit all datasets again, using the local polynomial regression method (Section 2). Figure 4 shows the fitted curves for datasets K101E+G190S, L74V+K101E+G190S, M230L, and M41L+K101E+G190S+.

T215Y. Fitted curves for datasets L74V+K101E+G190S and M41L+K101E+G190S+T215Y showed that the parametric and nonparametric methods gave comparable results.

As shown in Fig. 4, lower concentrations of EFV clearly stimulate the replication of HIV K101E+G190S and M230L compared to no EFV or at high EFV concentrations. However, this property cannot be recognized by using the traditional sigmoidal model fitting. To test the efficiency of our proposed nonparametric method when the monotonicity property is satisfied, we applied our method to the L74V+K101E+G190S and M41L+K101E+G190S+T215Y datasets. The dose response curves obtained by using the nonparametric model are very similar to those by the sigmoidal model, which confirms the efficiency of the nonparametric method. Table 3 compares the estimated IC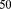 values for all datasets obtained by using both methods. When the sigmoidal model works well, as for HIV mutant strains G190S, K101E, L74V+K101E+G190S, M41L+K101E+G190S+T215Y, M41L+G190S+T215Y, M41L+K101E+G190S.

**Table 3:** IC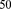 values estimated by using the nonparametric and the sigmoidal models for real datasets.

dataset	G190S	K101E	K101E+G190S	L74V+K101E+G190S	M41L+K101E+G190S+T215Y
Sigmoidal	58.5	13.80		2939.9	801.4
Nonparametric	71.84	13.06	3149.69	2808.16	914.29

dataset	M230L	M41L+G190S+T215Y	K101E+G190S(D10)	M41L+G190S+T215Y(D10)	M41L+K101E+G190S+T215Y(D10)
Sigmoidal		52.8		165.8	5394.8
Nonparametric	156.73	58.78	19330.61	195.92	5746.94

+T215Y(D10), and M41L+G190S+T215Y(D10) (these datasets also satisfy the monotonicity property, as shown in Table 2), the two estimated IC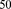 values for the same dataset are close (Table 3). In contrast, because of the lack of monotonicity, the sigmoidal model fails to fit the curves for HIV strains of K101E+G190S, M230L and K101E+G190S(D10) (Table 2). For these datasets, the nonparametric model becomes a better alternative for IC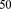 estimations (Table 3).

## Discussion

When the dose response relationship and associated parameters such as IC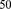 are studied, data observations, which make the pattern nonmonotonic, are generally deleted in order to use the model (1) or similar monotonic functions. However, by deleting these “unusual” observations, some important information may be lost. For example, in Fig. 2, the observation that a lower EFV concentration stimulates HIV K101E+G190S replication can be neglected if these data points are deleted. Removing the unusual data points leaves only 2 observations in this dataset, making the fitting procedure impossible.

In this paper, we proposed a nonparametric approach as an alternative to the parametric sigmoidal model to fit the dose response curve and estimate IC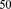, and suggested a monotonic check of the dose response relationship at the first stage. If the monotonicity is satisfied, either the traditional sigmoidal model fitting or our local polynomial regression fitting can be applied. If monotonicity is not satisfied, our model is more suited to estimate the IC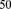. Using this new approach, important dose response features will not be omitted. A similar approach has been used to quantify protein lysate assays [16], although no monotonicity needs to be satisfied in that case.

Our proposed method can also be used for other dose response modeling scenarios, such as hormesis dose response curves. In toxicology, hormesis is a special dose response feature characterized by low dose stimulation and high dose inhibition [17]–[19], giving a J-shape dose response curve. Our nonparametric model is more suited than traditional monotonic models to fit this J-shaped curve. Also, our method can be used to model other nonparametric curves such as U-shaped dose response relationships frequently observed in toxicology and epidemiology studies [20].

The human trefoil peptide (TFF1), a small cysteine-rich secreted protein, stimulates cell migration by chemotaxis. The dose response curve of TFF1 inducing breast cancer cell movement shows a clear bell shape [21]. Similar curves are also seen in may other biomedical studies [22]–[27]. Our nonparametric model may be more suitable than sigmoidal models to fit such dose response curves and estimate the IC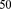.

Our approach for estimating IC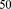 can also be used to estimate the half-maximal effective concentration, which is commonly used when the drug enhances its target's activity, and the lethal dose 50%, or the lethal concentration and time of a toxic substance or radiation represents the dose needed to kill half the tested population. Since the results we obtained are based on large sample theory, a potential limitation of our proposed method is that a moderate sample size may be needed, although a minimum sample size is not determined.
